# Pathways of intergenerational transmission of depression: The role of the Fast Track intervention

**DOI:** 10.1017/S0954579425100588

**Published:** 2025-09-10

**Authors:** Laura Gorla, W. Andrew Rothenberg, Jennifer Godwin, William E. Copeland, Conduct Problems Prevention Research Group

**Affiliations:** 1 Center for Child and Family Policy, Duke University, Durham, NC, USA; 2 University of Vermont, Burlington, VT, USA

**Keywords:** Depression, intergenerational relationships, mental health, preventive interventions

## Abstract

Although depression can be transmitted across generations, less is known about how this cycle can be interrupted. This study examines whether the multilevel Fast Track intervention (clinicaltrials.gov, NCT01653535) disrupts intergenerational transmission of depression. Children at high risk for aggression were randomly assigned to a 10-year control group or intervention targeting parenting and children’s intrapersonal, interpersonal, and academic skills. The original sample included 891 first-generation (G1) participants who reported on their depression and their children’s (second-generation; G2) internalizing problems. At age 34, 374 G2 participants (*n* = 191 intervention, *n* = 183 control) reported on their and their children’s (third-generation; G3) emotional difficulties. Mediated path models showed that a cascading model where higher G1 depressive symptoms influence higher G2 childhood depressive symptoms, leading to higher G2 adulthood depressive symptoms, which in turn is connected with greater G3 emotional difficulties, emerged only in the control group. The Fast Track intervention disrupted the pathways from G1 depressive symptoms to G3 emotional difficulties, from G2 childhood depressive symptoms to G2 adulthood depressive symptoms, and from G2 adulthood depressive symptoms to G3 emotional difficulties, highlighting the importance of preventive interventions in altering developmental trajectories of psychopathology.

## Introduction

Depression is among the most prevalent mental health disorders in the United States. In 2021 alone, according to the National Survey on Drug Use and Health (NSDUH), an estimated 21 million adults in the United States experienced at least one major depressive episode, with the highest rates observed in individuals aged 18–25. These numbers are even more worrying when looking at the prevalence of depressive symptoms among children and adolescents. According to the NSDUH, 4.5 million U.S. adolescents aged 12 to 17 had at least one major depressive episode in 2023.) In the United States, 1 in 5 children aged 3–17 suffers from a mental, emotional, behavioral, or developmental disorder (Centers for Disease Control and Prevention, 2024; McGorry et al., 2024).

Unsurprisingly, the widespread occurrence of depression carries a significant economic burden (König et al., 2020). In the United States, the cost associated with depression increased from $236.6 billion in 2010 to $326.2 billion in 2018 (Greenberg et al., 2021). Given these alarming statistics, gaining a better and deeper understanding of factors that exacerbate or hinder the onset of depression, as well as possible interventions aiming to reduce depression, is crucial. This necessity is even stronger because depression is highly associated with neurological and psychosocial impairments (Cambridge et al., 2018; Gorwood et al., 2008), poor health outcomes (Gao et al., 2015), hospitalizations (Frank et al., 2023), maladaptive parenting (Wolford et al., 2019), and child abuse (Windham et al., 2004). Moreover, given that depressive symptoms tend to vary in intensity, duration, and recurrence, sometimes starting in childhood or adolescence and becoming stable throughout life, several studies have focused on depression’s trajectories, long-term effects, and intergenerational transmission (Goodman, 2020; Musliner et al., 2016; Shore et al., 2018).

### Intergenerational transmission of depression

The concept that individuals whose parents have suffered from depressive symptoms have an increased risk for depression has been defined as the intergenerational transmission of depression (Goodman, 2020). The transmission of depression from grandparents to parents (first and second generation, G1–G2, respectively) and then from parents to children (third generation, G3) has been explained as a result of several factors, such as psychobiological and molecular mechanisms and the quality of the environment that the offspring of depressed parents experience (Ivanova et al., 2022; Sawyer et al., 2019). For instance, among the key mechanisms of intergenerational transmission of depression are a history of long-term exposure to stressors (Hammen et al., 2012; Rothenberg et al., 2018) and the use of harsh parenting practices within the family (Wolford et al., 2019), which are strongly associated with children’s internalizing problems.

Children of depressed parents face a heightened risk of developing depressive symptoms and are more likely to experience depression at an earlier age than their peers (Goodman & Garber, 2017; Gotlib et al., 2020; Jaffee et al., 2021; Josefsson et al., 2019). Moreover, they are more likely to have poorer and less adaptive cognitive functioning, be more vulnerable to higher levels of irritability and fear, not develop adequate coping skills for handling stress, and build more problematic interpersonal relationships (see Gotlib et al., 2023 for a review). All these characteristics represent strong risk factors, potentially disrupting the formation of supportive interpersonal relationships, decreasing academic success, increasing risks of substance use and social functioning impairments, and significantly increasing mental health difficulties across later developmental stages (LoParo et al., 2024).

Given the long-lasting effect of depression, it is not surprising that individuals with previous depressive symptomatology in their childhood and adolescence likely bring these experiences into their parenthood. Several studies have highlighted that parents who suffer from depression tend to diminish their capacity for self and other care, perceive parenting as a difficult task, experience high levels of parenting stress, are less sensitive toward their children’s needs, and use maladaptive parenting practices (Biaggi et al., 2016; Vreeland et al., 2019). All these aspects of parenting challenge children’s development, potentially starting a cascade of adverse effects and contributing to the transmission of depression across generations (Goodman, 2020; Pinto et al., 2020).

### Breaking the intergenerational transmission of depression

Given the extensive literature on the intergenerational transmission of depression, it is crucial to identify protective factors and understand how it is possible to reduce this intergenerational transmission. Depression does not happen in a vacuum but is always influenced by the social context in which individuals live. According to Bronfenbrenner’s (1979) developmental ecology theory, individuals belong to and are influenced by different environments that differ in their proximity to the child. Following this theory, certain social environment factors can mitigate or even disrupt the adverse effects of depression, both within and across generations. Among these, cognitive-behavioral preventive interventions acting at both individual, school and community, and population levels have been identified as an effective way to reduce risk factors associated with the transmission of maladaptive parenting, acting as a protective buffer against parents’ and children’s depressive symptoms (Bernaras et al., 2019; Ssegonja et al., 2019; Thapar et al., 2022). However, most studies have used cross-sectional data and explored only two-generation relationships, with few exploring how preventive and multilevel interventions have long-term and enduring effects on several generations (Gotlib et al., 2023; Hill et al., 2020).

We aim to fill this gap by examining whether participating in a multilevel preventive intervention - the Fast Track program (Conduct Problems Prevention Research Group [CPPRG]) – can interrupt the intergenerational transmission of depressive symptoms across three generations for children with early conduct problems. Initiated in the early 1990s, Fast Track is a comprehensive, multicomponent intervention designed to prevent the development of severe conduct problems in children identified by their parents and teachers as exhibiting early aggression. Grounded in the idea that comprehensive and continuous support from childhood to adolescence can have lasting benefits into adulthood, the intervention included parent management training for parents, child social-emotional and cognitive skills training, academic tutoring, home visits from grades 1 to 10, and a universal SEL program for the school in grades 1–5. Fast Track specifically aimed to foster both interpersonal and intrapersonal development, promoting prosocial skills, reducing antisocial and aggressive behaviors, and equipping children with problem-solving and emotion regulation strategies. The intervention also supported children’s emergent literacy skills, enhancing participants’ academic competence.

Multiple studies have documented the long-lasting impact of Fast Track, showing that the intervention significantly reduced original participants’ need for general and mental health services through age 18 (Jones et al., 2010) and had a positive impact on children’s mental health into adulthood (Dodge et al., 2015; Godwin & CPPRG, 2020; McCabe et al., 2025; Sorensen et al., 2016). As adults, Fast Track participants exhibited fewer externalizing and internalizing problems and were less likely to use corporal punishment with their own children (Sorensen et al., 2016). The intervention’s benefits have also extended into the third generation. Fast Track participants’ children were less likely to be born into maladaptive environments (Rothenberg et al., 2023) and to use inpatient and outpatient mental health services (Rothenberg et al., 2024).

### Current study

The current study advances the understanding of the intergenerational transmission of depression by investigating this process using a longitudinal and three-generation study whose sample was originally screened for overt behavioral problems. Specifically, it examines whether depressive symptoms in the first generation are associated with mental health difficulties (e.g., depressive symptoms in childhood and depressive symptoms in adulthood) in the second generation and whether these difficulties are, in turn, linked to emotional difficulties in the third generation. Moreover, the study examines whether participating in the Fast Track intervention disrupts the transmission of depression from one generation to another.

Several hypotheses guided the current study. First, we hypothesized that high levels of depressive symptoms in first-generation (G1) parents would predict greater depression in their children (G2) during childhood, which would, in turn, be associated with an increased rate of depressive symptoms in adulthood. Second, we expected that G2 depression in adulthood would serve as a risk factor for emotional difficulties in the third-generation (G3) children. While testing these hypotheses, we also tested for a cascading pathway in which elevated depression in G1 is linked to greater G2 depression during childhood, which is subsequently connected to high depression in adulthood, ultimately leading to poorer outcomes in G3s. We also hypothesized that for individuals who were randomly assigned to participate in the Fast Track intervention, this intergenerational transmission of depression would be disrupted, thereby promoting better mental health for the next generation, compared to the control group that did not receive the Fast Track intervention.

## Method

### Participants

Between 1991 and 1993, 55 elementary schools in Durham, NC; Nashville, TN; rural Pennsylvania; and Seattle, WA, that were considered “high risk” based on crime and poverty statistics of the neighborhoods they served, were selected for Fast Track participation. Within these schools, teachers screened 9,594 kindergarteners for aggressive behavior. Those children scoring in the top 40% within each cohort and site were then screened for home behavior problems by their parents. Then, teachers’ and parents’ screening scores were standardized and combined into a severity-of-risk screen score. Based on this score, high-risk children were selected. Recruitment began with the highest-scoring child, continuing until designated sample sizes were reached within sites, cohorts, and groups. Exclusion criteria were that the children’s parents did not speak English, the child was currently in foster care, or the families planned to move within 1 year. Within each site, the schools were divided into one to three paired sets of schools, and one set in each pair was randomly assigned to intervention and control conditions. 91% of recruited families agreed to participate, yielding a sample of 891 G2 children (intervention group, *N* = 445; control group, *N* = 446; see CPPRG, 2020 for further details). In 2020–2021, at age 34, the original G2 participants were invited to complete a follow-up survey. Of the 891 original participants, 848 were still alive (95%), and 568 (67% of the living sample) agreed to participate. Of these 568 participants, 398 (71% of all G2s who agreed to participate at age 34) met criteria for reporting on their family environment, as they a) had at least 1 G3 child under 18 years old, and b) either lived with the child at least 20% of the time or reported seeing or communicating with their child or the child’s custodial guardian more than once a month. Of these eligible participants, 374 (92% of eligible G2s; *n* = 191 intervention group, *n* = 183 control group) provided information on their G3 children, with parenting practices and linked mental health measures applied to a single randomly selected G3 per family (see Supplemental Figure 1 CONSORT diagram for detailed information).

As for sociodemographic characteristics, G1 parents who reported their own depressive symptoms and their G2 children’s depression when G2s were aged 6 – 11were, on average, aged 30.70 years at the first report (SD = 6.44). Their racial distribution was Black (48.51%), White (49.75%), and Other (1.74%), and they were predominantly women (97.51%). The G2’s mean age at the selection was 6.58 years (SD = 0.48), 69% were boys, and race varied (Black, 51%; white, 47%; other, 2%). G3s were mostly males (52%), mainly aged 9.37 years old (SD = 4.38, min-max = 2 – 17). The G2 parent subsample and initial G2 sample, as well as the G2 parent intervention and control groups, showed significant differences in only 4 out of 29 pre-treatment and demographic differences (see Supplemental Table 1). Specifically, G2 men and those with higher externalizing scores or social competence scores at age 6 were less likely to participate at age 34. Within the Age 34 intervention group, participants had lower friendship satisfaction scores and lower externalizing behavior risk scores but higher social competence scores at age 6 compared to those in the control group. Finally, as G1 depression scores were very close to the clinical depression cut-off of 16, we tested for differences in depression scores between our current sample and the Center for Epidemiological Studies – Depression scale (CES-D; Radloff, 1977) general population. We found that G1 depression was significantly higher than the general population (M_G1 depression_ = 16.07, M_general population_ = 7.94, SD_G1 depression_ = 8.4, SD_general population_ = 7.53; *t* = 18.70, df = 1823, *p* < .001; Cohen’s *d* = 1.05), highlighting that G1s in the current sample were at higher risk for depression.

### Procedures

In grades 1–5, all intervention families were provided 2-hour group interventions that included parent management training, children’s social skill training, parent-child enrichment sessions, and, in first grade only, reading tutoring. The sessions were weekly in grades 1, biweekly in grade 2, and monthly in the later grades. In addition, families received regular home visits to support their child’s learning and behavior, and children received academic tutoring at school, as needed (Bierman et al., 2017; McMahon et al., 1996). Furthermore, to promote children’s social and emotional competencies, a universal, teacher-implemented social-emotional learning curriculum was provided at all sites except for Durham, NC (where school mergers after grade 1 did not allow further implementation; Kusché & Greenberg, 2020). In grades 6–10, Fast Track intervention children received a middle school transition program, youth forums, and parent-youth groups on topics of adolescent development, such as alcohol, tobacco, drugs, and decision-making. Other topics that were targeted by the Fast Track intervention included positive involvement and monitoring, coping with peer pressure, romantic relationships, sex education, substance use, vocational opportunities, life skills, and summer employment (CPPRG, 2020; Greenberg et al., 2011). Parent and child participation in programing and implementation fidelity were high. Written consent from parents and oral assent from children were obtained, and all procedures were approved by the Institutional Review Boards of participating universities. The Fast Track randomized controlled trial is registered at clinicaltrials.gov, registry number NCT01653535. Instructions for requesting and using Fast Track data are available at https://fasttrackproject.org/requesting-and-using-data-2/.

Between the G2s’ ages of 6 and 15 (grades K-8), G1 parents annually reported their relationships with their G2 children, their parenting practices, and, occasionally, their relationships with their G1 parenting partners. G1 parents received compensation for participating in these interviews. When the original G2 participants reached age 34, they were invited to complete a survey assessing their relationship with their romantic partner (if applicable), their parenting of their G3 children, and their G3 children’s mental health. They received modest compensation for their time, completing surveys online via an emailed link, over the phone, or in person.

### Measures

#### Pre-intervention, demographic covariates, and intervention status

G3 age and sex, as well as other 29 pre-intervention and demographic covariates, were examined as potential control variables and are listed in Supplemental Table 1 (see www.fasttrackproject.org for more details; Bierman et al., 2004; CPPRG, Conduct Problems Prevention Research Group, 2007). However, controlling for all 29 covariates when analyzing G1, G2, and G3 associations simultaneously resulted in model under-identification. To address this issue, we used only covariates significantly associated with G2 or G3 measures in zero-order correlations (see figure notes below). Intervention status was coded 0 for control group members and 1 for intervention group members.

#### G1 depression

G1 depressive symptoms were measured using the Center for Epidemiological Studies – Depression scale (CES-D; Radloff, 1977). The CES-D is a 20-item self-report measure designed to measure current levels of depressive symptomatology. Each of the twenty items states an experience related to depression that the respondent may have had during the previous week and asks the respondent to select the value best describing how frequently the experience occurred. Responses are coded on a four-point Likert scale ranging from 0 to 3, including: “Rarely, or none of the time (less than 1 day),” “Some or a little of the time (1 – 2 days),” “Occasionally or a moderate amount of time (3 – 4 days)” and “Most or all of the time (5 – 7 days).” The CES-D does not have subscales but uses a total score to estimate depression and has a cut-off of 16 to identify individuals at risk for clinical depression. G1 parents completed the CES-D scale in years 1and 3 of the study when children were 6and 8 years old, and we created a total CES-D score by combining the mean of each year’s score. The instrument had high internal consistency in both years (*α* =.879 and *α* =.890).

#### G2 childhood depression

G2 childhood depressive symptoms were measured using the Child Behavior Checklist (CBCL; Achenbach, 1991; Achenbach & Rescorla, 2003). The scale identifies behavioral and emotional problems in children and adolescents by asking parents to describe their children within the past 6 months and comprises 112 items significantly differentiating clinically-referred from non-referred children. G1 parents completed the CBCL scores for their G2 children in years 1, 2, 3, 5, and 6 of the study when the children were 6, 7, 8, 10, and 11 years old. Following Achenbach’s (1991) guidelines, we created a G2 childhood depression score for each year by summing 12 items related to depressive symptoms (e.g., “deliberately harms self or attempts suicide,” “overtired without good reason”) and then created a total G2 childhood depression scale by combining the mean of each year’s score to create a single score. We found moderate to acceptable internal consistency in all years (*α*_y1_ = .60, *α*_y2_ = .63, *α*_y3_ = .60, *α*_y5_ = .71, *α*_y6_ = .70).

#### G2 adulthood depression

G2 adulthood depression was measured using the *Adult Self-Report* (Achenbach & Rescorla, 2003). This is a 132-item survey designed to assess the emotional and behavioral problems in adults in a standardized format. The participants are asked to think about the past 6 months and response options are “not true,” “sometimes true,” and “often true.” The instrument author’s aggregate externalizing T-score and internalizing T-score (based on national norms within gender; mean score = 50 [SD = 10]) were computed. G2 parents completed this instrument in years 19 and 26 of the study when they were 25 and 31 years old. We created a mean adulthood depression score (*α*_y19_ = .93, *α*_y25_ = .94).

#### G3 emotional Difficulties

G3 emotional difficulties were measured using G2 parents’ reports on the Strengths and Difficulties Questionnaire (SDQ; Goodman, 2001). The SDQ is a 25-item instrument capturing positive and negative attributes of children aged 2 to 17 years old and evaluating children’s mental health difficulties, dysfunctional behaviors, and attitudes within the context of socialization with peers and adults. Parents are asked to rate how much their children present or not a behavior/problem (0 = *Not true*; 1 = *Somewhat true*; 2 = *Certainly true*). Although the instrument includes items about emotional difficulties (e.g., “He has many worries or often seems worried”), conduct problems (e.g., “Often he lies or cheats”), hyperactivity/inattention (e.g., “He is restless, overactive, cannot stay still for long”), and problems with peers (e.g., “He is rather solitary, prefers to play alone”), we used only the emotional difficulties subscale given the focus of the current study. G2 parents completed the SDQ scale in year 28 of the study when G2 parents were 34 years old. Overall, higher scores indicate greater emotional difficulties (*α* =.70).

### Sensitivity analyses

To ensure the robustness of our results, we conducted several sensitivity analyses. For the sensitivity analysis, we also used G2 internalizing and externalizing problems in years 1, 3, 5, and 6 of the study when the children were 6, 8, 10, and 11 years old. Specifically, we used the CBCL for assessing G2 internalizing and externalizing problems. Following Achenbach’s (1991) guidelines, we created a total score of G2’s internalizing problems and a total score of G2’s externalizing problems by taking the mean of each year’s score. We found good internal consistency in all years for both internalizing (*α*_y1_ = .68, *α*_y2_ = .71, *α*_y3_ = .65, *α*_y5_ = .69, *α*_y6_ = .71) and externalizing problems (*α*_y1_ = .71, *α*_y2_ = .75, *α*_y3_ = .76, *α*_y5_ = .76, *α*_y6_ = .74).

### Analytic plan

We used R software (version 4.2.2., R Core Team, 2020) and the lavaan package (Rosseel, 2012) to perform a series of path analyses. To investigate study questions, we ran mediated path models with G3 emotional difficulties as the dependent variable, G1 depressive symptoms as the main predictor, and G2 childhood and adulthood depressive symptoms as mediators. Specifically, we ran path models in which G1 depressive symptoms predicted G2 childhood depressive symptoms, which predicted G2 adulthood depression, which in turn predicted G3 emotional difficulties. We also tested for the direct effects of G1 depressive symptoms on G3 emotional difficulties. These paths were analyzed in multiple group models to test whether they differed by intervention status. First, four paths were constrained to be equal across intervention and control groups. These paths included (1) G1 depressive symptoms predicting G2 childhood depression, (2) G2 childhood depressive symptoms predicting G2 adulthood depression, (3) G2 adulthood depressive symptoms predicting G3 emotional difficulties, and (4) G1 depressive symptoms predicting G3 emotional difficulties. Then, one by one, these paths were freed to vary across groups, and the difference in fit was compared with a 1-degree-of-freedom chi-square test. If the chi-square test revealed that the model fit better when the path was allowed to freely vary across groups, the path remained unconstrained. Otherwise, it remained equal across groups. This procedure allowed us to identify exactly which intergenerational pathways differed across the Fast Track intervention and control groups.

We included any of the 29 covariates that emerged as significant correlates in zero-order correlations with G2 or G3 measures in each path model. We used full information maximum likelihood to handle missing data and robust maximum likelihood estimation to account for skewness or kurtosis in study variables and to ensure intent-to-treat analytic frameworks were followed. Finally, we evaluated model fit using the chi-square (χ^2^) statistic and its p-value, Comparative Fit Index (CFI), Root Mean Square Error of Approximation (RMSEA) and its *p*-value, Standardized Root Mean Square Residual (SRMR), and Goodness of Fit (GIF). Following Kline’s (2023) recommendations, we considered acceptable fit to be a non-significant χ^2^, CFI > .90, RMSEA ≤ .08 with non-significant p-value, SRMR < .10, and GFI > .95. The current study was not preregistered. Materials and analysis code for this study are available by emailing the corresponding author.

## Results

**Figure 1.**
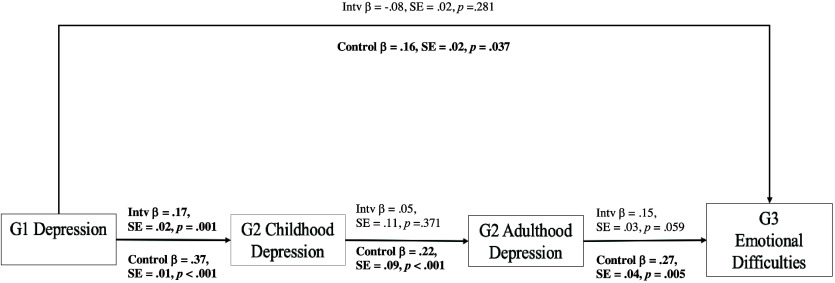
Path analysis: intergenerational transmission of depression and its impact on G3 emotional difficulties. χ^2^ (50) = 48.242, *p* = .54, CFI = 1.00, RMSEA = .00, *p* = 1.00, SRMR = .02, GIF = .99. *Note.* Bolded paths indicate *p* < .05. Intv = Fast Track intervention group. Control = Fast Track control group. G1 = generation 1, G2 = generation 2, G3 = generation 3. Several associations with covariates emerged in zero-order correlations that were controlled in the present model, but not presented in the figure due to space constraints. They were the associations between (1) G2 childhood depression and G2 adulthood depression with G1 family and friends satisfaction when G2s were 6, (2) G2 childhood depression with the school the G2 went to at age 6, (3) G2 childhood depression with G2 kindergarten stress scale when G2s were 6, (4) G2 childhood depression and G2 adulthood depression with G2 participation in the cohorts of Durham, North Carolina, and rural Pennsylvania, (5) G2 childhood depression and G2 adulthood depression with oppositional aggressive score when G2s were 6, (6) G2 childhood depression with G2 social competence score when G2s were 6, (7) G2 childhood depression with participation in the 1993 cohort of G2s, (8) G2 childhood depression and G2 adulthood depression with socioeconomic status when G2s were 6, (9) G2 childhood depression and G2 adulthood depression with G1 ethnicity, (10) G2 childhood depression with total aggression score standardized within cohort and school when G2s were 6, (11) G2 childhood depression with neighborhood questionnaire total score, (12) G2 adulthood depression and G3 emotional difficulties with G2 gender, (13) G3 emotional difficulties and G3 gender, (14) G3 emotional difficulties and G2 % hostile attributions when G2s were 6, (15) G3 emotional difficulties and G2 gender. Contact the corresponding author if interested in these covariate associations.

Table 1 reports descriptive statistics and zero-order correlations for the main study variables. The results summarize each path’s main findings and indirect effects. Figure 1 reports exact parameter estimates and final omnibus model fit statistics.

**Table 1.** Descriptive statistics and correlations of main study variables

	*N*	*M* (SD) or %	1.	2.	3.	4.
1. G1 Depression	403	16.07 (8.4)	1.00			
2. G2 Childhood Depression	403	2.66 (2.2)	**.37**	1.00		
3. G2 Adulthood Depression	400	5.94 (4.62)	**.13**	**.19**	1.00	
4. G3 Emotional Problems	374	1.57 (1.84)	.07	**.18**	**.23**	1.00

*Note.* Bold values are significant with *p* < .05.

After examining chi-squared difference tests, the model predicting G3 emotional difficulties fits best when all the paths were freed to vary across the intervention and control groups. This indicates that the magnitude of the pathways from G1 depressive symptoms to G2 childhood depressive symptoms, G2 childhood depressive symptoms to G2 adulthood depressive symptoms, G2 adulthood depressive symptoms to G3 emotional difficulties, and G1 depressive symptoms to G3 emotional difficulties varied across the control and the intervention group.

Specifically, in both the control and intervention groups, G1 depressive symptoms predicted G2 childhood depressive symptoms, with high depressive symptoms reported by G1 parents predicting more G2 depressive symptoms during childhood (Figure 1). Interestingly, higher G2 childhood depressive symptoms predicted higher G2adulthood depressive symptoms, and higher G2 adulthood depressive symptoms predicted greater G3 emotional difficulties only in the control group (Figure 1). In the treatment group, these paths were not significant. Furthermore, a direct association between G1 depressive symptoms and G3 emotional difficulties emerged only in the control group, where we found that high G1 depressive symptoms predicted G3 emotional difficulties, showing a connection between depressive symptoms across generations (Figure 1). By contrast, the association between G1 depression and G3 emotional difficulties was not present in individuals who participated in the Fast Track intervention (Figure 1). Moreover, the indirect effects differed between the two groups. The intergenerational mediating pathways from high G1 depressive symptoms to high G2 childhood depressive symptoms to greater G2 adulthood depressive symptoms were significant only in the control group (G2 Adulthood Depression Indirect Effect = .042, SE = 0.01, 95% CI = [.02, .06], *β* = .08, *p* < .001), but not in the intervention group (G2 Adulthood Depression Indirect Effect = .004, SE = .01, 95% CI =[−.01,.02], *β* = .01, *p* = .388).The intergenerational mediating pathways from high G2childhood depressive symptoms to greater G2 adulthood depressive symptoms to more G3 emotional difficulties were moderately significant in the control group (G3 Emotional Difficulties = .05, SE = .02, 95% CI = [.01,.09], *β* = .06, *p* = .023), but not in the intervention group (G3 Emotional Difficulties = .006, SE = .01, 95% CI = [−.01, .02], *β* = .01, *p* = .450). The total intergenerational mediating pathways were significant only in the control group (Total Indirect Effects = .12, SE = .03, 95% CI = [.06, .18], *β* = .29, *p* < .001), but not in the intervention group (Total Indirect Effects = −.01, SE = .02, 95% CI = [−.05, .03], *β* = − .07, *p* = .725).

### Sensitivity analyses

To further examine the intergenerational transmission of depression on G3 emotional difficulties, we conducted a series of sensitivity analyses.

Given the strong connection highlighted in the literature between depression, internalizing, and externalizing problems, we explored the impact of G2 internalizing and G2 externalizing problems in childhood, along with G2 internalizing problems in adulthood, on G3 emotional difficulties. Specifically, we ran two additional multigroup path models. In the first, G1 depressive symptoms predict G2 childhood internalizing problems, which, in turn, predict G2 adulthood internalizing problems, ultimately leading to G3 emotional difficulties. In the second, G1 depressive symptoms predict G2 childhood externalizing problems, which, in turn, predict G2 adulthood depression, ultimately leading to G3 emotional difficulties.

Supplemental Table 2 shows the results. The first model fits best when all the paths were allowed to vary and revealed very similar patterns of results observed in the main depression model. Specifically, in the control group only, we identified a cascading model in which G1 depression predicted G2 childhood internalizing problems, which were significantly related to G2 adulthood internalizing problems, ultimately leading to G3 emotional difficulties. Furthermore, a significant direct path from G1 depression to G3 emotional difficulties also emerged in the control group. Conversely, among participants who received the Fast Track intervention, the only significant paths were the ones from G1 depression to G2 childhood internalizing problems and from G2 childhood internalizing problems to G2 adulthood internalizing problems. These findings further support our main depression model, underscoring the protective effects of the Fast Track intervention in disrupting several intergenerational paths.

**Figure 2.**
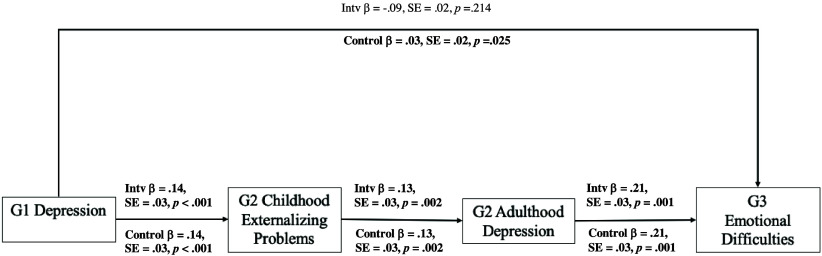
Sensitivity analyses. Intergenerational transmission of depression on G2 childhood and adulthood externalizing problems and G3 emotional difficulties. χ^2^ (73) = 73.901, *p* = .45, CFI = .99, RMSEA = .01, *p* = 1.00, SRMR = .02, GIF = .99. *Note.* Estimates from (1) G1 depression to G2 childhood externalizing problems, from (2) G2 childhood externalizing problems to G2 adulthood depression, and from (3) G2 adulthood depression to G3 emotional difficulties are equal across intervention and control groups due to equality constraints in the model. Bolded paths indicate *p* < .05. Intv: Fast Track intervention group. Control = Fast Track control group. G1 = generation 1, G2 = generation 2, G3 = generation 3. Several associations with covariates emerged in zero-order correlations that were controlled in the present model, but not presented in the figure due to space constraints. They were the associations between (1) G2 childhood externalizing problems with G1 family and friends satisfaction when G2s were 6, (2) G2 childhood externalizing problems with physical punishment mean score when G2s were 6, (3) G2 childhood externalizing problems with G2 kindergarten stress scale when G2s were 6, (4) G2 childhood externalizing problems and G2 adulthood depression with socioeconomic status when G2s were 6, (5) G2 childhood externalizing problems and G2 adulthood depression with oppositional aggressive score when G2s were 6, (6) G2 childhood externalizing problems and warm, harsh, and appropriate discipline mean when G2s were 6 (7) G2 childhood externalizing problems with G2 participation in the cohorts of Durham, North Carolina, Nashville, Tennessee, and rural Pennsylvania, (8) G2 childhood externalizing problems with participation in the 1992 and 1993 cohort of G2s, (9) G2 childhood externalizing problems with G2 ethnicity, (10) G2 childhood externalizing problems, G2 adulthood depression and G3 emotional difficulties with G2 gender, (11) G2 childhood externalizing problems with G2s social competence score when G2s were 6, (12) G2 childhood externalizing problems and G2 adulthood depression with neighborhood questionnaire total score, (13) G2 childhood externalizing problems and G2 adulthood depression with total aggression score standardized within cohort and school when G2s were 6, (14) G3 emotional difficulties and G3 gender, (15) G3 emotional difficulties and G2 % hostile attributions when G2s were 6. Contact the corresponding author if interested in these covariate associations.

The second model fits best when all the paths (except for the pathway from G1 depression to G3 emotional difficulties) were constrained to be equal across the control and intervention groups (Figure 2). This indicates no significant difference between control and intervention groups in most intergenerational paths. Interestingly, only in the control group, did the path from G1 depression to G3 emotional difficulties remain significant. These findings suggest that the transmission patterns observed for depression and internalizing problems do not extend to externalizing problems, highlighting a specificity in how emotional difficulties are passed down across generations.

## Discussion

Using data from a longitudinal, three-generation study, we investigated the intergenerational transmission of depression, its impact on the third generation’s emotional adjustment, and the role of the Fast Track intervention as a potential disruptor of these intergenerational pathways. Overall, our findings support a cascading pathway in which depressive symptoms in the first generation predict emotional difficulties (i.e., childhood and adulthood depression) in the second generation, which in turn lead to greater emotional difficulties in the third generation. Interestingly, the Fast Track intervention changed some cascading pathways.

Our first hypothesis, that high G1 depressive symptoms would be linked to greater G2 childhood depressive symptoms, which in turn would be connected to higher G2 adulthood depressive symptoms, was supported. In both the control and intervention groups, G1 depression emerged as a risk factor for childhood depression in G2, aligning with a robust body of research showing the adverse effects of parental depression on children’s mental health (Goodman & Garber, 2017; Ivanova et al., 2022; Jaffee et al., 2021; Josefsson et al., 2019). Importantly, these effects extended beyond childhood and into adulthood, reinforcing evidence that individuals with experiences of emotional and mental health difficulties in childhood are more vulnerable to suffering from depression later in life (Biaggi et al., 2016; Goodman, 2020; Gotlib et al., 2023; Vreeland et al., 2019). However, the progression from childhood depression to adult depression was observed only in the control group, with participants in Fast Track not exhibiting the same pattern.

Previous research has demonstrated that Fast Track significantly contributed to developing interpersonal and prosocial skills, enhanced social problem-solving abilities, and reduced antisocial behaviors and conduct problems (Bierman et al., 2004; CPPRG, Conduct Problems Prevention Research Group, 2007, 2011). By promoting social and self-regulation skills in childhood and early adolescence, Fast Track has shown a positive and long-lasting impact on G2’s well-being (Godwin & CPPRG, 2020; Rothenberg et al., 2023; Sorensen et al., 2016). Specifically, at age 25, individuals who received the intervention exhibited lower rates of externalizing, internalizing, and substance use problems, reported higher levels of overall well-being, and were less likely to be convicted of violent and drug crimes and engaged in risky sexual behavior compared to their peers in the control group (Dodge et al., 2015). These positive outcomes persisted into early adulthood as, at age 31, the intervention group continued to show reduced internalizing problems and greater personal strengths due to indirect intervention effects (McCabe et al., 2025). Not surprisingly, the current study confirmed the positive effects of the Fast Track intervention on depressive symptoms and emotional difficulties, showing that G2 childhood depression did not develop into G2 adulthood depression for those receiving the intervention. These positive and lasting effects may be attributed to the intervention’s promotion of adaptive intrapersonal skills, such as emotion regulation strategies and effective coping mechanisms. These socio-emotional abilities promote children’s healthy development as they support children in perceiving the environment as less hostile, responding more appropriately to external and internal stimuli, perceiving less anxiety and depression, and building positive, functional, and meaningful significant relationships (Daniel et al., 2020; Domitrovich et al., 2017; Extremera & Rey, 2015).

Our second hypothesis, that G2 depressive symptoms in adulthood would be linked to greater emotional difficulties in G3 children, was supported but only in the control group. In control families, G2 adulthood depressive symptoms emerged as a risk factor for emotional problems in G3s, aligning with previous literature showing long-lasting, longitudinal, and intergenerational adverse effects of parents’ depression (Goodman, 2020; Musliner et al., 2016; Shore et al., 2018). As suggested by the strong associations between depressive symptoms across the first, second, and third generations in the control group, evidence emerged for a cascading pathway of high depression risk and transmission. Specifically, in the control group, elevated depressive symptoms in G1 were linked to greater G2 depressive symptoms during childhood, which was subsequently connected to high depression in adulthood, ultimately resulting in poorer emotional outcomes in G3s. Furthermore, in the control group, G2 depressive symptoms in childhood significantly mediated the adverse impact of G1 depression on G2 adult depression, being consistent with prior research on the developmental course and intergenerational transmission of depression (Goodman, 2020; Musliner et al., 2016; Shore et al., 2018). Notably, both the direct path from G2’s adulthood depression to G3’s emotional difficulties and the path from G2’s childhood depression to G3’s emotional difficulties throughout the G2’s adulthood depression mediation were significant only within the control group, suggesting that participation in the Fast Track intervention may have halted the intergenerational and longitudinal transmission of depression by buffering against the emergence of depression in G2 during adulthood. The long-lasting effects of the Fast Track intervention may be attributed to its intensive and extensive nature. Grounded in the premises that comprehensive and continuous support from childhood through adolescence can have enduring benefits into adulthood, the Fast Track intervention provided targeted support to children, parents and teachers during key developmental periods, particularly critical for the onset of depression and its association with adverse long-term outcomes (Copeland et al., 2021). Moreover, given the Fast Track intervention positive effects in building interpersonal skills, G2 parents’ ability to carry through their use of intrapersonal skills into adulthood may have promoted more adaptive family environments characterized by less G2 depression and corporal punishment (Dodge et al., 2015; Rothenberg et al., 2024), therefore making children’s emotional difficulties less likely.

Finally, the significant pathway from G1 depressive symptoms to G3’s emotional difficulties, observed exclusively among individuals who did not receive the Fast Track intervention, suggested that emotional difficulties may be uniquely positioned to be transmitted across three generations, establishing a distinct pathway of risk continuity. This specificity of emotional difficulties transmission across generations was also suggested by both the presence and absence of cascading pathways from G1 depression to G3outcomes via G2 internalizing and externalizing problems, respectively. Given that depression is closely linked to difficulties in emotion regulation and positive emotion expression (Vanderlind et al., 2020), early experiences of depression in childhood may hinder individuals’ ability to understand, express, and manage emotions later in life, particularly when experiencing demands and stressors related to their role as adults and parents. As a result, these individuals may be less capable of responding sensitively to their children’s emotional needs, potentially increasing nonsupportive parenting behaviors. Additionally, individuals with a history of depression may model maladaptive ways of coping with life events, changes, stress, and conflicts, unintentionally exposing their children to dysfunctional interpersonal patterns. Over time, this exposure may increase the next generation’s emotional difficulties (Nyquist & Luebbe, 2022). Although these aspects collectively provide support for the spillover effects in which emotional difficulties experienced in childhood create conditions for later emotional difficulties, not all children whose parents suffer from depression develop depression symptomatology, requiring a deep exploration of individual pathways to understand which specific moderators and mediators might alter the intergenerational transmission of depression. For instance, dimensions related to parenting (e.g., behavioral control and autonomy granting), child characteristics (e.g., personality and sensitivity), the relationships between the two parents (e.g., co-parenting, romantic attachment), and contextual factors (e.g., peer relationships, social support, family income) might play a protective or risk role in the depression developmental trajectories. Given the relevance of alternative pathways and mechanisms, future research on how preventive, intense, and multilevel interventions such as the Fast Track intervention would act on these dimensions would be particularly beneficial.

Closely connected to this, a distinct pathway of risk continuity between internalizing and externalizing pathways emerged in the current study. These findings might appear surprising, considering that the Fast Track intervention initially targeted children at high risk for aggressive behaviors. However, a possible explanation may lie in the high levels of depressive symptoms reported by G1, which slightly exceed the clinical cut-off and were higher than those typically observed in the general population. Given that G1parentsin the current sample were at higher risk for depression, their G2 children may be more likely to exhibit increased internalizing behaviors by modeling their parents’ behaviors (Bandura, 1969), as well as later transmit these psychopathological patterns to their G3 children, a trend that is consistent with homotypic intergenerational continuity of psychopathology transmission (Goodman et al., 2011; Marceau et al., 2022).

Finally, two important considerations should be taken into account when interpreting our findings: potential reporter bias and the specific mechanism of action and components of the Fast Track intervention. Indeed, a long-standing debate in the literature explored the extent to which parents can accurately assess their children’s internalizing and externalizing symptoms. Several studies have shown that, overall, parents tend to have a low-to-moderate accuracy in reporting their children’s internalizing symptoms, which are typically less visible, less evident, and more ambiguous than externalizing symptoms (De Los Reyes & Kazdin, 2005). These biases might be especially pronounced when parents suffer from depressive symptoms themselves (Kroes et al, 2003). All these aspects align with the depression-distortion hypothesis, which posits that depression promotes a negative bias in parental perceptions of their children’s behavior and emotional problems (Richters & Pellegrini, 1989). Although it is important to acknowledge that, due to G1s’ high levels of depression, their sensitivity towards their children’s depressive symptoms might have been heightened, more recent evidence has begun to challenge this vision, finding only small effects of parental depression on their reporting of youth psychopathology (Olino et al., 2021). Moreover, the significant association between G2 childhood depression (reported by G1 parents) and G2 adulthood depression (self-reported by G2s), partially challenges the depression-distortion hypothesis and points to a stable and consistent pattern of depressive symptoms and emotional difficulties across time. Linked to this, the absence of this association in the intervention group underscores the potential protective role of specific components of the Fast Track intervention. Among these (see CPPRG, 2020, for a detailed description), G2 participants received the PATHS® (Promoting Alternative Thinking Strategies) Curriculum (Greenberg et al., 2011), a social-emotional learning intervention targeting prosocial skills, self-control, emotional awareness and understanding, and social problem solving, all dimensions often compromised in individuals suffering from depression symptoms. It is possible that improved emotion recognition and coping strategies fostered by the intervention significantly improved overall self-esteem and confidence of G2s across time, making them less susceptible to parenting stress and psychopathological symptoms in adulthood. Furthermore, as the Fast Track intervention actively involved both families and schools and fostered communication and dialogue inside and outside the family, it is also possible that G2s developed a more positive vision of the broader social context, creating a more optimistic perceptions of their world and increasing their sense of security and confidence in recognizing and reporting their children’s symptomatology accurately (McCabe et al., 2025).

The study also has limitations. First, although G1 and G2 depressive symptoms were collected at several time points, G3 emotional difficulties were collected only at one time point, potentially limiting more complex longitudinal analyses. Second, for analytical purposes, G1 and G2 depressive symptom scores collected at each time point were combined into a single composite score. While this approach suited the current study’s aims, it might not have caught depression changes over time. Further research is needed to explore the trajectories and temporal changes of intergenerational transmission of depression, to test how depression duration, severity, and recurrence are transmitted across generations, and to explore the potential effects of the Fast Track intervention against these aspects. Third, G2’s childhood depression scale presented moderate internal consistency. Fourth, both G2’s childhood depression and G3’s emotional difficulties were reported by parents rather than by children themselves, increasing reporter bias. Despite these limitations, this study makes a significant contribution by demonstrating that depression is not confined to a single generation but can be transmitted from one generation to another, especially through the mediation of internalizing problems in childhood. However, these pathways can be changed by a multilevel preventive intervention. These findings highlight the importance of programs and policies to support childhood interventions to increase family members’ well-being and reduce emotional difficulties, which can pay dividends to children at the time of the intervention as well as when they become adults and even into the next generation. The intensive, preventive, and multilevel nature of the Fast Track intervention underscores the public relevance of investing in such programs, with the idea that supporting the well-being of today’s families can yield long-lasting benefits, positively influencing future generations. Notably, preventive interventions are effective in reducing the onset and severity of mental health problems as they enable early detection and promote resilience in individuals, ultimately reducing the population demand for mental health services. Collectively, these aspects contribute to a significant decrease in healthcare costs, a consideration that is particularly relevant given the widespread prevalence of depression, as well as its associated economic and societal burden. Given that supporting evidence-based interventions that operate across different levels (e.g., individual, family, school, and community) may reduce present disparities in mental health care access and outcomes, the intergenerational effects observed in the current study further underscore the societal relevance of investing in the current generations to build a healthier future for next ones.

## Conclusion

Although there is a substantial body of research on the intergenerational transmission of depression, few studies have examined this topic using a longitudinal, three-generation intervention sample. The current study advances understanding of the intergenerational transmission of depression by examining whether depressive symptoms in the first generation are associated with mental health difficulties in the second generation and whether these difficulties are, in turn, linked to mental health challenges in the third generation. Moreover, the study tests whether participating in the Fast Track intervention disrupts these intergenerational pathways.

Taken together, current results suggest several main conclusions. First, G1 depressive symptoms emerged as a risk factor for G2 depressive symptoms in childhood, which in turn led to higher G2 adulthood depressive symptoms. Importantly, the pathway from childhood to adult depressive symptoms was observed only in the control group, supporting the Fast Track intervention’s protective role against longitudinal development of depression. Second, in the control group only, G2 depression in adulthood was associated with greater emotional difficulties in G3 children, highlighting that the Fast Track intervention was effective in breaking the negative effects of G2 adulthood depressive symptoms on the next generation’s emotional well-being. Finally, the specificity of the intergenerational transmission of depressive symptoms and emotional difficulties emerged, showing a direct effect of G1 depressive symptoms on G3 emotional difficulties only in the control group. These results underscore that emotional difficulties may be uniquely positioned to be transmitted across three generations, making a distinct pathway of risk continuity that can be broken with early intervention.

## Supporting information

10.1017/S0954579425100588.sm001 Gorla et al. supplementary material 1 Gorla et al. supplementary material

10.1017/S0954579425100588.sm002 Gorla et al. supplementary material 2 Gorla et al. supplementary material

## Supplementary material

The supplementary material for this article can be found at https://doi.org/10.1017/S0954579425100588.
