# Supplementary material for: Pathways of intergenerational transmission of depression: The role of the Fast Track intervention
Source: Dev Psychopathol. 2025 Sep 10:1–11. Online ahead of print. doi: 10.1017/S0954579425100588 (PMC12486471; doi:10.1017/S0954579425100588)
Supplement: Gorla et al. supplementary material 1 — Gorla et al. supplementary material [file S0954579425100588sup001.docx]

**Supplemental Figure 1.** CONSORT Flow Chart (Adapted from CPPRG, 2020)

G2s Assessed for eligibility at school (*n*=9,594)

Excluded (*n*=6,320)

Not meeting inclusion criteria (*n*=5,994)

Refused to participate (*n*=326)

G2s Assessed for eligibility at home (*n*=3,274)

Excluded (*n*=2,383)

Not meeting inclusion criteria (*n*=2,249)

Refused to participate (*n*=75)

Did not matriculate to 1st grade (*n*=59)

G2s Randomized (*n*=891)

G2s Assigned to receive intervention (*n*=445)

Received intervention (*n*=445)

G2s Assigned to control condition (*n*=446)

G2s Lost to interview at age 34 (*n*=158)

Scheduled interview but never completed (*n*=10)

Declined to participate (*n*=29)

Could not locate (*n*=29)

Started interview but not enough data (*n*=11)

Deceased (*n*=26)

In Prison (*n*=23)

Agreed to participate but won’t complete (*n*=11)

Found but never responded (*n*=3)

Withdrew from the study (*n*=16)

G2s Lost to interview at age 34 (*n*=165)

Scheduled interview but never completed (*n*=8)

Declined to participate (*n*=32)

Could not locate (*n*=43)

Started interview but not enough data (*n*=13)

Deceased (*n*=17)

In Prison (*n*=22)

Agreed to participate but won’t complete (*n*=4)

Found but never responded (*n*=11)

Withdrew from the study (*n*=15)

G2s Agreed to participate at age 34 (*n*=281)

G2s Agreed to participate at age 34 (*n*=287)

G2s Didn’t participate in parenting survey (*n* = 92)

No living children (*n*=76)

All children older than 18 (*n*=1)

Unknown amount of child contact (*n*=1)

Number of children unknown (*n*=3)

Refused to participate (*n*=11)

Participated but did not complete parenting or G3 mental health measures (*n =*6)

G2s Didn’t participate in parenting survey (*n* =78)

No living children (*n*=70)

All children older than 18 (*n*=1)

Unknown amount of child contact (*n*=3)

Refused to participate (*n*=4)

Participated but did not complete parenting or G3 mental health measures (*n =*18)

G2s Participated in parenting survey (*n*=183)

G2s Participated in parenting survey (*n*=191)
