# Supplementary material for: Pathways of intergenerational transmission of depression: The role of the Fast Track intervention
Source: Dev Psychopathol. 2025 Sep 10:1–11. Online ahead of print. doi: 10.1017/S0954579425100588 (PMC12486471; doi:10.1017/S0954579425100588)
Supplement: Gorla et al. supplementary material 2 — Gorla et al. supplementary material [file S0954579425100588sup002.docx]

**Pathways of Intergenerational Transmission of Depression: The Role of the Fast Track Intervention**

**Supplementary Materials**

**Supplementary Table S1**

Pre-Intervention & Demographic Means for the G2 Parent Subsample, G2 Non-Parent Sample, G2 Intervention Parent Subsample and G2 Control Parent Subsample by Intervention Status

|  | Original Sample | | | | Age 34 Parent Sample | | | |
| --- | --- | --- | --- | --- | --- | --- | --- | --- |
| Variable | Participated in Age 34 Parent Sample  (*n* = 398) | | Did Not Participate in Age 34 Parent Sample  (*n* = 493) | | G2 Intervention Parents  (*n* = 191) | | G2 Control Parents  (*n* = 183) | |
|  | *M or %* | *SD* | *M or %* | *SD* | *M or %* | *SD* | *M or %* | *SD* |
| Pre-Intervention Variables Measured in G1-G2 Family Before Start of Fast Track Intervention in 1991-1993 | | | | | | | | |
| Depression Score* | 16.31 | 9.75 | 16.33 | 10.45 | 16.54 | 9.72 | 16.07 | 9.81 |
| % Hostile Attributions | 68% | 26% | 66% | 25% | 69% | 26% | 67% | 26% |
| Aggressive Behavior Score | 22.04 | 8.14 | 22.61 | 8.24 | 22.37 | 8.47 | 21.68 | 7.77 |
| Appropriateness Score | 3.52 | 0.81 | 3.60 | 0.71 | 3.55 | 0.76 | 3.49 | 0.86 |
| Family Satisfaction | 2.17 | 0.72 | 2.12 | 0.72 | 2.14 | 0.76 | 2.21 | 0.68 |
| Friendship Satisfaction | 2.37 | 0.61 | 2.31 | 0.61 | **2.28^b^** | 0.67 | **2.46^b^** | 0.51 |
| Physical Punishment Mean Score | 0.22 | 0.22 | 0.22 | 0.24 | 0.20 | 0.21 | 0.24 | 0.23 |
| Kindergarten Stress Scale | 5.20 | 4.18 | 5.33 | 4.04 | 5.30 | 4.30 | 5.09 | 4.06 |
| Verbal Punishment Mean Score | 0.25 | 0.31 | 0.26 | 0.32 | 0.26 | 0.34 | 0.25 | 0.17 |
| Socioeconomic Status | 24.09 | 12.08 | 24.61 | 13.16 | 24.33 | 12.40 | 23.84 | 11.75 |
| Oppositional Aggressive Score | 0.25 | 0.17 | 0.27 | 0.18 | 0.25 | 0.16 | 0.25 | 0.17 |
| Warm, Harsh, and Appropriate Discipline Mean | 2.00 | 0.28 | 2.00 | 0.27 | 2.00 | 0.28 | 2.00 | 0.28 |
| Social Competence Total Score | **2.09^a^** | 0.59 | **2.01^a^** | 0.58 | **2.15^b^** | 0.58 | **2.03^b^** | 0.61 |
| Letter and Word Identification Score | 12.45 | 3.96 | 12.75 | 4.96 | 12.75 | 4.22 | 12.12 | 3.64 |
| Total Number Correct on Emotion Recognition Questionnaire | 10.70 | 2.71 | 10.72 | 2.88 | 10.66 | 2.69 | 10.74 | 2.74 |
| Warmth | 3.58 | 0.75 | 3.58 | 0.82 | 3.64 | 0.75 | 3.51 | 0.75 |
| Mean % of Competent Responses to Social Problem Solving Scale | 62% | 22% | 63% | 22% | 62% | 22% | 61% | 22% |
| Neighborhood Questionnaire Total Score | -0.04 | 0.62 | -0.04 | 0.59 | -0.07 | 0.65 | -0.01 | 0.58 |
| Average Standard Wechsler Intelligence Scale for Children Score | **-0.14^a^** | 0.74 | **-0.02^a^** | 0.82 | -0.11 | 0.77 | -0.17 | 0.71 |
| Externalizing Behavior Risk Standardized Score | **0.82^a^** | 0.63 | **0.94^a^** | 0.71 | **0.75^b^** | 0.62 | **0.89^b^** | 0.63 |
| Demographic Variables | | | | | | | | |
| % Black | 50% |  | 52% |  | 52% |  | 47% |  |
| % Member of Cohort 1 | 36.75% |  | 33.20% |  | 36.41% |  | 37.11% |  |
| % Member of Cohort 2 | 35.50% |  | 36.46% |  | 36.41% |  | 34.54% |  |
| % Member of Cohort 3 | 27.75% |  | 30.35% |  | 27.18% |  | 28.35% |  |
| % Male | **58.25%^a^** |  | **78.41%^a^** |  | 61.65% |  | 54.64% |  |
| % From Durham, NC Site | 27.25% |  | 22.40% |  | 26.21% |  | 28.35% |  |
| % From Nashville, TN Site | 23.75% |  | 27.49% |  | 24.76% |  | 22.68% |  |
| % From Penn State, PA Site | **29.75%^a^** |  | **21.59%^a^** |  | 27.18% |  | 32.47% |  |
| % From Seattle, WA Site | **19.25%^a^** |  | **28.51%^a^** |  | 21.84% |  | 16.49% |  |

a Data represent significant difference (*p* < .05) between G2s who participated in age 34 parent sample and G2s who did not participate in age 34 parent sample.

b Data represent significant difference (*p* < .05) between G2 intervention parents and G2 control parents.

* represents that parental depression is one of the 29 covariates always analyzed while exploring the Fast Track intervention effects but excluded in this study because we used G1 depression as our main predictor.

**Supplementary Table S2.** G2 Internalizing and Externalizing models results

| **Model 1**: G1 Depression 🡪 G2 Childhood Internalizing Problems 🡪 G2 Adulthood Internalizing Problems 🡪 G3 Emotional Difficulties | | | | | | | | | | |
| --- | --- | --- | --- | --- | --- | --- | --- | --- | --- | --- |
|  | **Control group** | | | | | **Intervention group** | | | | |
|  | B | SE | 95% CI | β | *p* | B | SE | 95% CI | β | *p* |
| G1 depression 🡪 G2 childhood internalizing problems | .23 | .03 | [.16, .29] | .33 | **<.001** | .09 | .03 | [.03, .15] | .15 | **.005** |
| G2 childhood internalizing problems 🡪 G2 adulthood internalizing problems | .46 | .14 | [.19, .73] | .18 | **.001** | .36 | .14 | [.08, .65] | .12 | **.011** |
| G2 adulthood internalizing problems 🡪 G3 emotional difficulties | .03 | .01 | [.01, .05] | .25 | **.003** | .01 | .01 | [-.01, .02] | .06 | .469 |
| G1 depression 🡪 G3 emotional difficulties | .03 | .02 | [.003, .07] | .16 | **.031** | -.01 | .02 | [-.04, .02] | -.06 | .427 |
| Indirect effects G2 childhood internalizing problems | .10 | .03 | [.04, .17] | .06 | **.002** | .03 | .02 | [-.00, .07] | .02 | .072 |
| Indirect effects G2 adulthood internalizing problems | .02 | .01 | [.001, .03] | .05 | **.037** | .00 | .004 | [-.01, .01] | .01 | .498 |
| Total indirect effects | .15 | .04 | [.07, .23] | .27 | **<.001** | .02 | .03 | [-.03, .07] | .01 | .389 |
| χ^2^ (64) = 57.631, *p* = .70, CFI = 1.00, RMSEA = .09, *p* = 1.00, SRMR = .02, GIF =.99 | | | | | | | | | | |
| **Model 2**: G1 Depression 🡪 G2 Childhood Externalizing Problems 🡪 G2 Adulthood Depression 🡪 G3 Emotional Difficulties | | | | | | | | | | |
|  |  | **Control group** | | | | **Intervention group** | | | |  |
|  | B | SE | 95% CI | β | *p* | B | SE | 95% CI | β | *p* |
| G1 depression 🡪 G2 childhood externalizing problems | .13 | .03 | [.08, .18] | .14 | **<.001** | .13 | .03 | [.08, .18] | .14 | **<.001** |
| G2 childhood externalizing problems 🡪 G2 adulthood depression | .08 | .03 | [.03, .13] | .13 | **.002** | .08 | .03 | [.03, .13] | .13 | **.002** |
| G2 adulthood depression 🡪 G3 emotional difficulties | .08 | .03 | [.03, .13] | .21 | **.001** | .08 | .03 | [.03, .13] | .21 | **.001** |
| G1 depression 🡪 G3 emotional difficulties | .04 | .02 | [.004, .06] | .17 | **.025** | -.02 | .02 | [-.05, .01] | -.09 | .214 |
| Indirect effects G2 childhood externalizing problems | .01 | .00 | [.003, .02] | .02 | **.009** | .01 | .00 | [.003, .02] | .02 | **.009** |
| Indirect effects G2 adulthood internalizing problems | .01 | .00 | [.001,  .013] | .03 | **.025** | .01 | .00 | [.001,  .013] | .03 | **.025** |
| Total Indirect Effect | .05 | .02 | [.02, .09] | .24 | **.002** | -.003 | .02 | [-.04, .03] | -.05 | .876 |
| χ^2^ (73) = 73.901, *p* = .45, CFI = .99, RMSEA = .01, *p* = 1.00, SRMR = .02, GIF =.99 | | | | | | | | | | |

Note. Table reports the unstandardized estimates (B), standard error (SE), the 95% confidence intervals of the unstandardized estimates, the standardized coefficients (beta), and the p-values.
